# SORT maneuver: ease and safety for the practitioner and the patient

**DOI:** 10.1186/s13613-020-00778-1

**Published:** 2020-11-30

**Authors:** Mahdi Najafi

**Affiliations:** 1 Department of Medical Biophysics, Western University, Room M402, Medical Sciences Building, 1151 Richmond St, London, ON N6A 3K7 Canada; 2 Department of Anesthesiology, Tehran Heart Center, Tehran University of Medical Sciences, Tehran, Iran

I read with interest the report of a randomized clinical trial by Sanaie et al. on the comparison of SORT maneuver versus a conventional technique of neck flexion lateral pressure (NFLP) for nasogastric tube (NGT) insertion in ICU-admitted patients [1]. This publication provides evidence for a greater ease of NGT insertion using SORT. Moreover, the success rate was much higher with SORT as compared to NFLP. The study looks flawless in methodology and straight forward in reporting the outcome results with a clever categorization for ease of insertion. Nevertheless, a general drawback is a lack of standard definitions for the favorable outcome like the time span attributable to “ease of insertion” and the determinants for “insertion failure”.

With regard to safety, they showed that the rate of complications was less in SORT compared to the other technique even though the difference was not significant. The power of study for this purpose, however, seems insufficient and a larger sample size is required to obtain a significant difference. Moreover, compared to the other complications studied, bleeding is more serious and more complex for which a kind of grading or scoring may be helpful for delineation of the severity.

A highlight from this study was that SORT is easy to be learnt by unskilled healthcare providers. I suggest the measurement of learning curve to support this claim. Then, it would be served as an advantage in demanding situations such as COVID-19 outbreak where trained personnel are overwhelmed by ICU overload [2]. The authors correctly mentioned the low number of providers and not including pediatric and high-risk groups as limitations. I’d like to add two other suggestions: studying hemodynamic response and comparing SORT with techniques that use equipment [3]. Hemodynamic compromise is a concern in cardiac and critically ill patients because of increasing myocardial oxygen demand. Patients with uncontrolled hypertension are even at greater risk [4]. The risk also theoretically increases when we insert NGT using laryngoscope with/without Magill forceps or using glidescope. SORT maneuver is devoid of both due to its smooth process and anatomical approach [5, 6]. These two proposals are among the issues to be addressed by future research projects on SORT maneuver.

## Data Availability

Not applicable.
